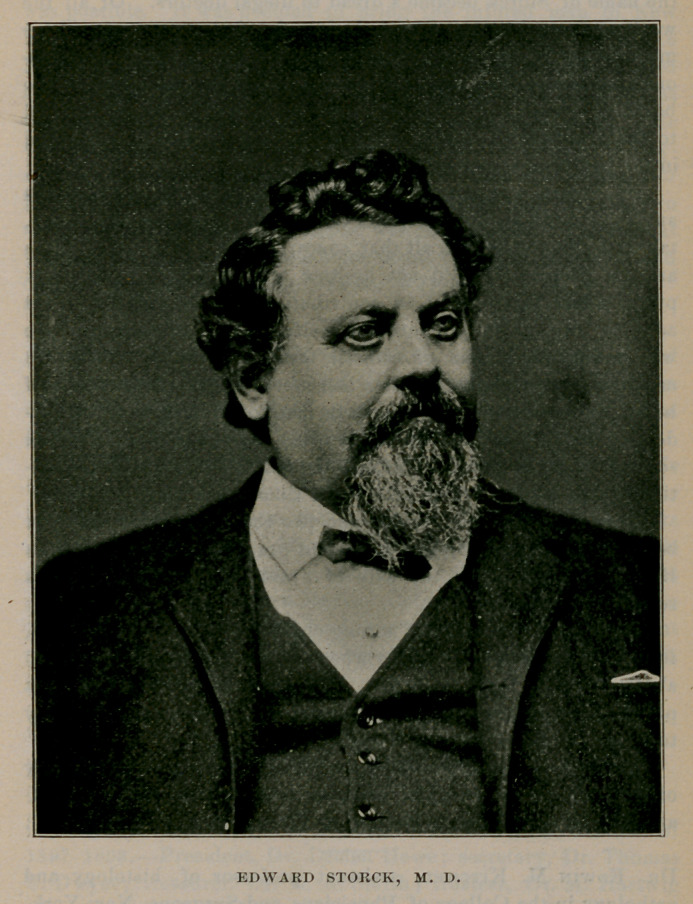# Dr. Edward Storck

**Published:** 1897-09

**Authors:** 


### Obituary.

Dr. Edward Storck, of Buffalo, died at his residence 220 East Eagle street, July 26, 1897, aged 66 years. He was born at Baden, South Germany, April 19, 1831, received his education in German and French schools, and afterward studied medicine with his father. In 1848 the revolution in South Germany interfered with the further prosecution of his studies, after which the family emigrated to America, settling in Williamsville, Erie county, New York. Edward Storck graduated from the University of Michigan in 1854, and in the same year married Lucy Grove. He then located in Buffalo, where he continued to reside until his death.

Dr. Storck soon acquired an extensive practice, principally among the German-American residents of Buffalo, and arose to prominence. When the civil war broke out Dr. Storck became a member of the Union defense committee, an organisation that contributed to the raising of troops for the field by supplying all the necessary means and methods necessary before the state or general government assumed control of the regiments. It was headed by Mayor Franklin A. Alberger, chairman, and made up of prominent citizens, among whom were Dr. Edward Storck, James Adams, Isaac Holloway, Alderman A. A. Howard and others. Dr. Storck easily became one of the most active and prominent men on the committee, and his enthusiasm, coupled with a vigorous young manhood, was such that he was enabled to perform an enormous amount of work. He espoused the Union cause loyally and worked faithfully to the end that the spirit of rebellion might be annihilated.

In 1872 Dr. Storck was elected president of the common council, which made him ex-officio president of the board of health, and under his energetic influence measures were adopted that soon controlled an epidemic of small-pox, then threatening to scourge the city. The first plans for an intercepting sewer, which involved means for the abatement of the Hamburg canal, were submitted by him to the common council in 1871, at which time he represented the fourth ward as alderman. He became a member of the Medical

Society of the County of Erie in 1854, and was elected to the presidency in 1878. He was chosen chairman of the board of censors in 1880, serving in that capacity for twelve consecutive years.

EDWARD STORCK, M. D.

During this period he was instrumental in securing legislation for the regulation of the study and practice of medicine, taking a prominent part in all the legislation of that stormy period. While he was chairman of the board of censors he fearlessly made war on quackery in all forms, and by his bold and energetic action drove unlawful practitioners from the City of Buffalo and the County of Erie. He was the inveterate foe of all shams and pretensions and the name of Storck became a dread to illegal doctors. Of all the work performed by Dr. Storck during the forty-two years of his membership in the Society none will stand out in more conspicuous prominence than that connected with his chairmanship of the board of censors. For this heroic service he will be forever entitled to the gratitude of community and his memory will be enshrined in the hearts of a grateful people.

Dr. Storck was not, however, simply a physician. There was another side to his character and career. He was a man of affairs, taking a deep interest in all that concerned the welfare of Buffalo and its people. He did not hesitate to take active part in any public affair that offered to contribute to the prosperity of his adopted city, or to promote the welfare of its inhabitants. He was, moreover, a man of esthetic tastes, interesting himself in music and art, contributing time and money to the advancement of both. In evidence of this it may be mentioned that he was president of the Buffalo Liedertafel for many years, and he also served several terms as president of the German Young Men's Association, which latter office he held at the time of his death.

He is survived by his widow and two children, one of the latter being Dr. Eugene E. Storck, at present practising his profession in Buffalo, and the other, the wife of Frank T. Williams, is also a resident of this city.

Dr. Storck was a man of strong character, amiable disposition, forceful in method and manner when necessary, gentle betimes, and always honest, frank and just. He acquired prominence as a physician, conspicuousness as a citizen and leaves a sweet memory to a large circle of devoted friends.

His funeral was attended by the Medical Society of the County of Erie and a large group of business men, friends and acquaintances. His remains were interred at Forest Lawn.

## Figures and Tables

**Figure f1:**